# Polystyrene-*block*-poly(ethylene oxide) copolymers as templates for stacked, spherical large-mesopore silica coatings: dependence of silica pore size on the PS/PEO ratio

**DOI:** 10.3762/bjnano.7.137

**Published:** 2016-10-14

**Authors:** Roberto Nisticò, Giuliana Magnacca, Sushilkumar A Jadhav, Dominique Scalarone

**Affiliations:** 1 University of Torino, Department of Chemistry, Via P. Giuria 7, 10125 Torino, Italy,; 2 NIS and INSTM Reference Centre, Via P. Giuria 7, 10125 Torino, Italy

**Keywords:** block copolymers, controlled porosity, mesoporous silica, soft templating, sol–gel

## Abstract

Large-mesopore silica films with a narrow pore size distribution and high porosity have been obtained by a sol–gel reaction of a silicon oxide precursor (TEOS) and using polystyrene-*block*-poly(ethylene oxide) (PS-*b*-PEO) copolymers as templates in an acidic environment. PS-*b*-PEO copolymers with different molecular weight and composition have been studied in order to assess the effects of the block length on the pore size of the templated silica films. The changes in the morphology of the porous systems have been investigated by transmission electron microscopy and a systematic analysis has been carried out, evidencing the dependence between the hydrophilic/hydrophobic ratio of the two polymer blocks and the size of the final silica pores. The obtained results prove that by tuning the PS/PEO ratio, the pore size of the templated silica films can be easily and finely predicted.

## Introduction

Mesoporous materials with large, tunable porosity are currently being investigated as selective molecular sieves, finding potential applications in many fields such as catalysis, encapsulation of proteins, filtration and separation of large molecules, membrane technology, drug delivery, dosing, adsorption, sensing, among many others [1–5].

Different approaches have been applied in order to obtain porous materials characterized by a controlled porous architecture. A sol–gel process, carried out in combination with a templating method and spin-coating deposition, is the easiest and the most versatile way for preparing well-organized, mesoporous, thin films. Among all the different templating approaches, the most widely used for the synthesis of nanostructured porous and high surface area oxides are soft templating (endotemplating) and hard templating (exotemplating) [6–7].

In general, templating agents arrange in the surrounding environment in such a way to generate a porous system in the newly synthesized materials, which replicates the 3D structure of the template arrangement. Often, both shape and size of the resulting porous system are directly and clearly correlated to the adopted porogen, but sometimes the template behavior and its effect on pore generation remain unclear.

Concerning soft templating, this procedure is related to the use of amphiphilic, low molecular weight surfactants or supramolecular cooperative macromolecules, which are relatively flexible in shape and size since they operate as structure directing agents in solution [8].

Amphiphiles are particular molecules containing a hydrophilic part (head) and a hydrophobic chain (tail). In solution and at certain conditions (i.e., when their concentration is higher than the critical micellar concentration, CMC) amphiphiles can spontaneously self-organize into well-defined supramolecular aggregates (host) which can be classified as normal and reverse micelles, emulsions, vesicles or liquid crystal phases and can shape or pattern other materials (guest), forming spherical nanostructures, rod-like short cylinders, lamellar sheets or worm-like structures [9–16].

Since these colloidal aggregates are in equilibrium with the isolated species (i.e., amphiphilic molecules or macromolecules and ions) present in the solution, their formation (and stability) is concentration dependent. In addition to the amphiphile concentration, the morphology and size of both micelles and micellar aggregates also depend on other solution parameters, such as the type of solvents, the solvent/nonsolvent ratio, the presence of additives, and on molecular parameters, such as the amphiphilic nature, molecular weight and architecture.

In the past twenty years, block copolymer-templated silica with large, spherical, accessible mesopores were intensively investigated [17–19] and several morphologies with different pore shapes and sizes were obtained by modulating the reactant ratio in the synthetic formulation as well as by changing the amphiphile parameters (e.g., number of polar/apolar moieties, functional groups, block length) [20–22].

Pluronics, nonionic amphiphiles consisting of poly(ethylene oxide) (PEO) and poly(propylene oxide) (PPO) triblock copolymers (PEO-*b*-PPO-*b*-PEO) [23], are among the most widely used soft templating agents to produce mesoporous silica. The templating process is based on the well-known attractive interaction between the silanol groups at the silica surface and PEO moieties [24]. In addition to Pluronics, other PEO containing amphiphilic block copolymers, such as polystyrene-*block*-poly(ethylene oxide) (PS-*b*-PEO), have been successfully employed to synthetize mesoporous oxides [19,25].

Starting from these previous works, we decided to develop a systematic study to gain better control on the morphological features of templated silica films. Thus, by fixing the TEOS/PS-*b*-PEO weight ratio of the micellar solutions in order to get spherical micelles (that is for TEOS/PS-*b*-PEO weight ratios of 95/5 or 93/7), we studied the effect of the PS/PEO ratio on the pore size of the templated silica films.

## Results and Discussion

Hybrid TEOS/block copolymer films obtained by spin-coating deposition of block copolymer micellar solutions were transformed into silica nanoporous thin layers by thermal treatment performed in air. This way, the organic templating species (i.e., block copolymers) were degraded and removed, leaving voids which formed the ordered nanoporous network [19].

In order to have the same final porous nanostructure (i.e., stacked spherical pores), the weight ratio between the silica precursor and polymeric soft templates was fixed at 95/5 and 93/7. In fact, as reported by other authors [26–27] and in our previous works [19,25], by further reducing the TEOS/block copolymer weight ratio, a change in the pore shape from spherical to elongated cavities is achieved as a result of the supramolecular arrangement of PS-*b*-PEO chains around a line instead of a dot (i.e., sphere-to-cylinder transition).

**Figure 1:**
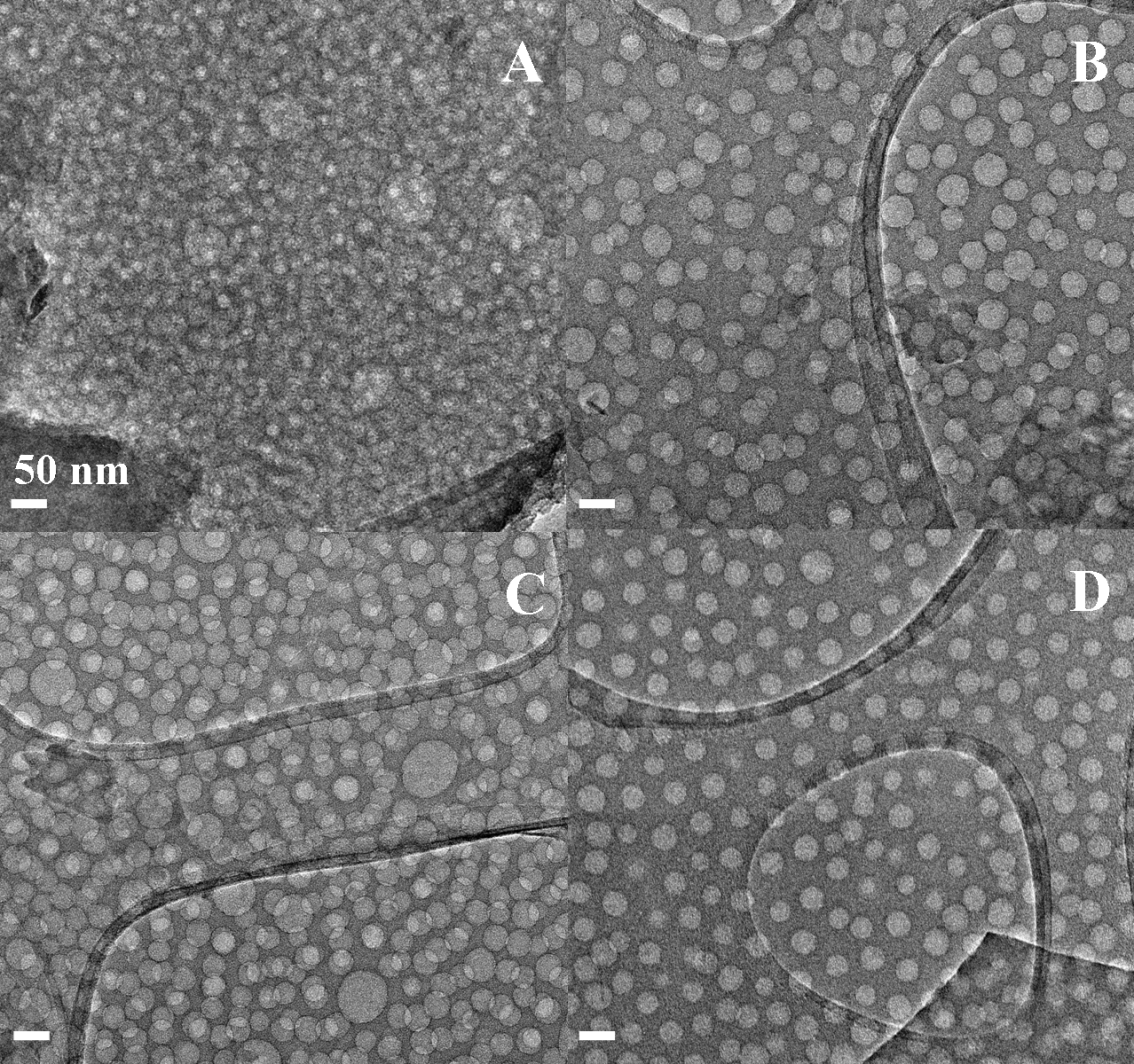
HRTEM micrographs of calcined 95TEOS/5PS-*b*-PEO films obtained by using different block copolymers, namely: PS_117_-*b*-PEO_543_ (A), PS_183_-*b*-PEO_145_ (B), PS_308_-*b*-PEO_250_ (C), and PS_567_-*b*-PEO_704_ (D). All micrographs were collected at the same magnification. Panel (B) reprinted with permission from [19], copyright 2014 Elsevier.

**Figure 2:**
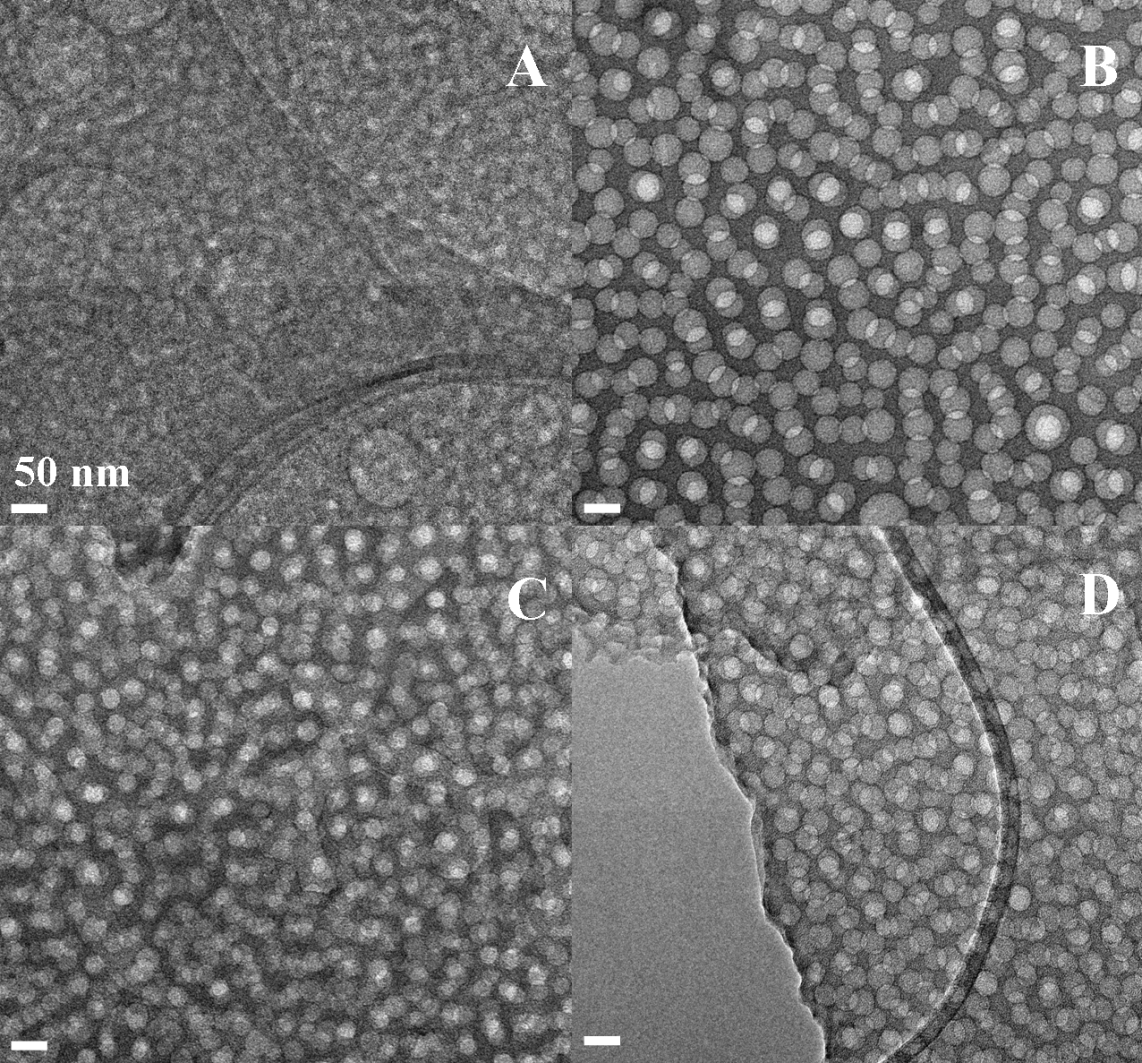
HRTEM micrographs of calcined 93TEOS/7PS-*b*-PEO films obtained by using different block copolymers, namely: PS_117_-*b*-PEO_543_ (A), PS_183_-*b*-PEO_145_ (B), PS_308_-*b*-PEO_250_ (C), and PS_567_-*b*-PEO_704_ (D). All micrographs were collected at the same magnification. Panel (B) reprinted with permission from [19], copyright 2014 Elsevier.

**Figure 3:**
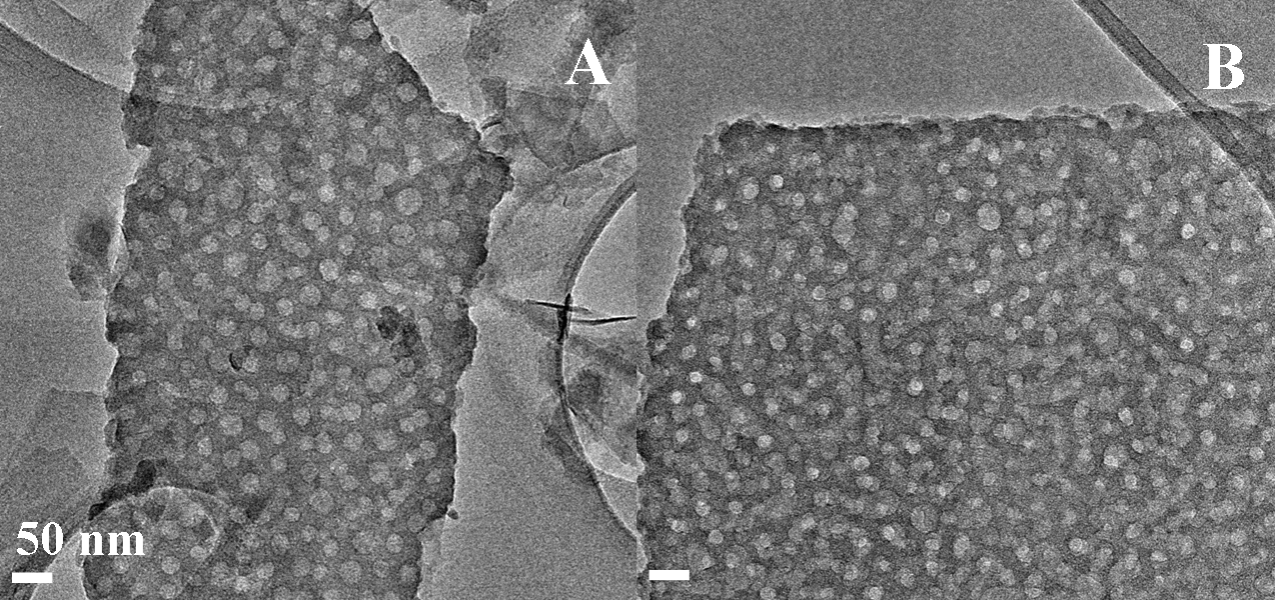
HRTEM micrographs of calcined TEOS/PS_120_-*b*-PEO_318_ films obtained at different TEOS/PS-*b*-PEO ratios, namely: 95/5 (A) and 93/7 (B). All micrographs were collected at the same magnification.

In this study, four PS-*b*-PEO copolymers with different size and block lengths (i.e., PS_117_-*b*-PEO_543_, PS_183_-*b*-PEO_145_, PS_308_-*b*-PEO_250_, and PS_567_-*b*-PEO_704_) were selected. HRTEM micrographs showing the mesoporous silica films prepared by soft templating of the four PS-*b*-PEO copolymers are reported in Figure 1 (95TEOS/5PS-*b*-PEO) and Figure 2 (93TEOS/7PS-*b*-PEO). Moreover, a fifth block copolymer (PS_120_-*b*-PEO_318_) was taken as a validation sample and HRTEM micrographs of both formulations (i.e., 95/5 and 93/7) are reported in Figure 3. Additionally, the average pore sizes calculated for each mesoporous silica film are collected in Table 1.

**Table 1:** Average pore size calculated from HRTEM measurements.

PS-*b*-PEO	PS/PEO ratio	95TEOS/5PS-*b*-PEO^a^	93TEOS/7PS-*b*-PEO^a^

PS_117_-*b*-PEO_543_	0.51	19 ± 2	17 ± 2
PS_120_-*b*-PEO_318_	0.89	25 ± 5	22 ± 4
PS_183_-*b*-PEO_145_	2.97	39 ± 5	41 ± 5
PS_308_-*b*-PEO_250_	2.91	36 ± 6	31 ± 4
PS_567_-*b*-PEO_704_	1.90	35 ± 3	34 ± 3

^a^Diameters are reported in nm ± standard deviation.

Independently from the length of the block copolymer used as a soft-templating agent, the TEM images confirm the formation of a homogeneous, internally porous system along the thickness of mesoporous films. The samples with the highest TEOS/block copolymer weight ratio (95/5) present stacked spherical pores, homogeneous in size, but are not regularly distributed. Reducing the TEOS/block-copolymer weight ratio to 93/7, and consequently decreasing the hydrophilic/hydrophobic solvent ratio, the spherical pore morphology is still retained, but a general improvement of the lateral order of the porous network is reached, thus also increasing the pore density [19]. In general, the pore diameters were found to be between approximately 20 nm up to 40 nm, with good reproducibility (i.e., low standard deviation).

**Figure 4:**
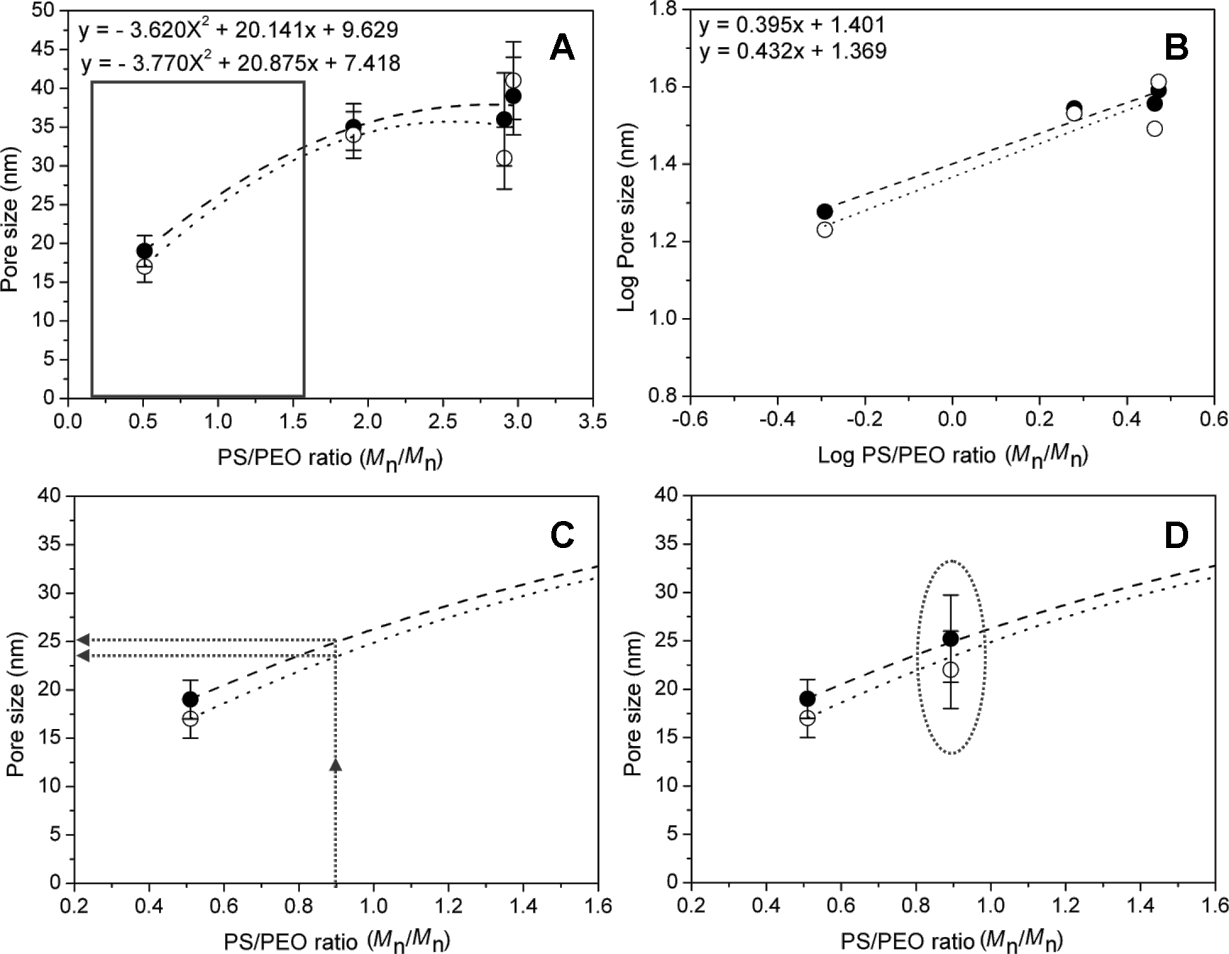
Evaluation of the pore size (nm ± SD) as a function of the PS/PEO ratio (A, C, D) and logarithm of pore size vs logarithm of the PS/PEO ratio (B) for the 95TEOS/5PS-*b*-PEO (black circles) and 93TEOS/7PS-*b*-PEO (white circles) samples. In particular, the equations in panels A and B refer, from top to bottom, to the 95/5 (dashed line) and 93/7 (dotted line) experimental fitting curves, respectively. Reliability of regressions: *R*^2^ > 0.98 and 0.83 (A), *R*^2^ > 0.96 and 0.88 (B). Black square in panel A is the zoomed area reported in panel C. Calculations of the pore size for the sample with 0.89 PS/PEO ratio are reported in panel C (dotted arrows), whereas experimental values are reported in panel D (dotted oval).

Moreover, since the four block copolymers selected present different block lengths, the effect of the block copolymer composition on the pore dimensions of the silica coatings was investigated. In detail, trying to rationalize the dependence of the pore size from the length of each block (both PS and/or PEO), no experimental trends were evidenced. Surprisingly, a second-order polynomial trend, with good data accuracy (Figure 4A), was achieved by plotting the average pore size as a function of the PS/PEO ratio. Both the investigated formulations (namely 95TEOS/5PS-*b*-PEO and 93TEOS/7PS-*b*-PEO) showed this polynomial trend. In particular, the lower the PS/PEO ratio (PS/PEO < 0.5), the smaller the pore size (i.e., micelle diameter) in the final templated materials. For a higher PS/PEO ratio (PS/PEO > 1) the pore diameter tends to reach a plateau. Furthermore, by making a linearization of such behavior in a decimal logarithmic scale (base 10), a linear trend with good data accuracy was also obtained (Figure 4B).

In order to validate this behavior, the theoretical pore size of the validation sample with a PS/PEO ratio of 0.89 was calculated by substituting this value in the four equations reported in Figure 4A,B. The calculated values are 25 nm (polynomial curve) and 24 nm (linear curve) for the 95/5 formulation and 23 nm (polynomial curve) and 22 nm (linear curve) for the 93/7 one. Concerning the polynomial curves, the pore size calculation is highlighted in Figure 4C, whereas experimental values calculated for these two formulations are reported in Figure 4D (numerical values collected in Table 2). Experimentally, 95TEOS/5PS_120_-*b*-PEO_318_ films present a pore size of 25 ± 5 nm and 93TEOS/7PS_120_-*b*-PEO_318_ films of 22 ± 4 nm, thus confirming the good matching between experimental and theoretical data.

**Table 2:** Compositions (wt %) of micellar solutions.

Sample name^a^	TEOS	PS-*b*-PEO^b^	Benzene	Ethanol	HCl

95TEOS/5PS-*b*-PEO	12.38	0.65	64.52	20.92	1.53
93TEOS/7PS-*b*-PEO	9.67	0.73	72.07	16.34	1.19

^a^Sample names refer to the composition (wt %) excluding solvents. ^b^Five different types of commercial PS-*b*-PEO block copolymers were selected: PS_117_-*b*-PEO_543_; PS_120_-*b*-PEO_318_; PS_183_-*b*-PEO_145_; PS_308_-*b*-PEO_250_; PS_567_-*b*-PEO_704_.

This dependence of the pore size, and of micelle size in the micellar solutions, may be explained by a different swelling effect of the PS core that depends on the micelle morphology.

In the investigated formulations, PS blocks form the internal core of the templating micelles and PEO blocks form the external corona. For low PS/PEO ratios, the PS-made micellar core occupies a smaller volume and is surrounded by a thicker PEO polar corona [28]. Due to the high incompatibility of PEO with the major component of the solvent mixture (i.e., benzene), in the micellar solution, PEO blocks are collapsed on the PS core, forming a dense layer that hinders a dynamic diffusion of benzene from the solution to the PS core, thus reducing the swelling and size of the micelle core. Vice versa, if the PEO block is shorter than the PS one, by increasing the PS/PEO ratio, the PEO corona becomes less dense, facilitating the diffusion of benzene through it. As a consequence, the PS-made micellar core increases up to a constant value. This happens when a swelling equilibrium with the apolar solvent inside/outside the templating micelles is achieved.

Even though further studies are necessary to better clarify this point, the interesting trends reported here confirm the importance of the template choice in order to obtain the desired porosity. In particular, based on the study presented here, it is possible to rationalize the design of the coating procedure and to control the final pore size in large-mesopore oxidic thin films by choosing the block copolymer template with the proper block length.

## Conclusion

A systematic analysis of the pore size of the block copolymer-templated mesoporous silica by varying the template structure has been realized. In particular, the templates selected in this study are PS-*b*-PEO polymers. By fixing the ratio between the block copolymer and TEOS (i.e., the silica precursor), stacked spherical porous systems were obtained. Five different PS-*b*-PEO copolymers were investigated and the importance of the PS/PEO ratio in order to control and predict the pore size in the final porous materials was proved. Until now many attempts have been made to control the size and shape of pores in templated silica, and in general, a direct relationship is sought between the size of micelles or pores and the length of the blocks. However, this is a simplification of the driving forces governing soft templating. The results here presented confirm that by fixing the composition of the micellar solution (i.e., the geometry of the system), a key parameter which drives the porosity is the hydrophilic/hydrophobic block ratio. Therefore, this study allows a rationalization of the preparation of mesoporous architectures in inorganic thin functional coatings.

## Experimental

### Synthesis and preparation of the mesoporous silica thin layers

Mesoporous silica thin films were synthesized by a sol–gel reaction of tetraethyl orthosilicate (TEOS, ≥ 99.0%, Aldrich) in ethanol (≥ 95.0%, Carlo Erba Reagents) under acidic conditions (HCl 37 wt %, Fluka Chemika) as reported in [19]. Different sol–gel solutions were prepared with a TEOS/HCl molar ratio of 3.5 (Table 2). All chemicals were used without further modification. Solutions were stirred at room temperature (rt) for 30 min using a magnetic stirrer. Five different types of commercial PS-*b*-PEO copolymers (PS_117_-*b*-PEO_543_ with *M*_n_ = 12,200-*b*-23,900; PS_120_-*b*-PEO_318_ with *M*_n_ = 12,500-*b*-14,000; PS_183_-*b*-PEO_145_ with *M*_n_ = 19,000-*b*-6,400; PS_308_-*b*-PEO_250_ with *M*_n_ = 32,000-*b*-11,000; PS_567_-*b*-PEO_704_ with *M*_n_ = 59,000-b-31,000) were purchased from Polymer Source Inc. (Dorval, Canada). Benzene (≥ 99.7%, Riedel-de-Haën) was chosen as a solvent for the preparation of copolymer solutions. Copolymer benzene solutions (1 wt %) were stirred for 30 min to ensure complete dissolution of the copolymer. Micellar solutions were prepared by adding the desired amount of sol–gel solution to the copolymer solution. Final solutions were spin-coated onto mica sheets of 1.27 cm × 1.27 cm × 15 mm, at 1000 rpm for 20 s, using an 8” Desktop Precision Spin Coating System, model P-6708D vs. 2.0. After deposition, the films were dried in a hood at rt for at least 12 h in order to reach complete evaporation of solvents. Hybrid films were then transformed into silica nanostructured thin layers by thermal treatment in a furnace under air atmosphere (400 °C for 2 h, ramp of 2 °C/min). The samples were named *X*TEOS/*Y*PS-*b*-PEO, where *X* and *Y* are the TEOS and block copolymer weight ratio excluding solvents. Two different weight ratios were analyzed: 95/5 and 93/7.

### Physicochemical characterization

High-resolution transmission electron microscopy (HRTEM) was used to evaluate the pore size and morphology of mesoporous silica films after the removal of the polymer templates. Micrographs were obtained by using a JEOL 2010 instrument (300 kV) equipped with a LaB_6_ filament. For the specimen preparation, a few drops of water were poured on the supported silica layer. After a few seconds the surface was gently scratched and the functionalized layer separated from the support. Fragments were then transferred onto holey carbon coated copper grids by lifting the grids onto the water layer. Pore sizes and distributions were calculated by using the software Particule2 on an average of 100 pores.
